# Scalable Fabrication of Black Phosphorous Films for Infrared Photodetector Arrays

**DOI:** 10.1002/advs.202403182

**Published:** 2024-07-21

**Authors:** Alexander Corletto, Purevlkham Myagmarsereejid, Shifan Wang, Wei Yan, Sivacarendran Balendhran, Huan Liu, Yu Lin Zhong, Kenneth B. Crozier, Munkhbayar Batmunkh, James Bullock

**Affiliations:** ^1^ Department of Electrical and Electronic Engineering The University of Melbourne Parkville Melbourne Victoria 3010 Australia; ^2^ Queensland Micro‐ and Nanotechnology Centre School of Environment and Science Griffith University Nathan Campus Brisbane Queensland 4111 Australia; ^3^ School of Physics The University of Melbourne Parkville Melbourne Victoria 3010 Australia; ^4^ ARC Centre of Excellence for Transformative Meta‐Optical Systems The University of Melbourne Parkville Melbourne Victoria 3010 Australia

**Keywords:** 2D materials, black phosphorous, electrochemical exfoliation, infrared photodetectors, scalable, thin films

## Abstract

Bulk black phosphorous (bP) exhibits excellent infrared (IR) optoelectronic properties, but most reported bP IR photodetectors are fabricated from single exfoliated flakes with lateral sizes of < 100 µm. Here, scalable thin films of bP suitable for IR photodetector arrays are realized through a tailored solution‐deposition method. The properties of the bP film and their protective capping layers are optimized to fabricate bP IR photoconductors exhibiting specific detectivities up to 4.0 × 10^8^ cm Hz^1/2^ W^−1^ with fast 30/60 µs rise/fall times under λ = 2.2 µm illumination. The scalability of the bP thin film fabrication is demonstrated by fabricating a linear array of 25 bP photodetectors and obtaining 25 × 25 pixel IR images at ≈203 ppi with good spatial fidelity. This research demonstrates a commercially viable method of fabricating scalable bP thin films for optoelectronic devices including room temperature‐operable IR photodetector arrays.

## Graphical abstract

Black phosphorous (bP) exhibits excellent potential for infrared photodetectors. However, most bP photodetector demonstrations are limited in scale, being fabricated from single flakes. Here, a scalable, solution‐based fabrication method for bP films is reported, which enables the demonstration of infrared photodetector arrays (25‐pixel linear arrays) with detectivities up to 4.0 × 10^8^ cm Hz^1/2^ W^−1^ and fast 30/60 µs rise/fall times.

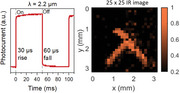

## 1 Introduction

The layered 2D black phosphorous (bP) crystal exhibits excellent infrared (IR) absorption, IR emission, and electronic transport properties, making it a promising material for advanced IR optoelectronics.^[^ ^1^, ^2^, ^3^, ^4^, ^5^, ^6^, ^7^, ^8^, ^9^, ^10^, ^11^, ^12^, ^13^, ^14^, ^15^, ^16^, ^17^, ^18^ ^]^ Black phosphorous has a direct bandgap that is tunable from 2.0 eV in monolayer form to 0.3 eV in bulk form which makes it suitable for use as the absorbing layer in short‐wave IR (SWIR) and mid‐wave IR (MWIR) photodetectors.^[^ ^19^, ^20^, ^21^ ^]^ Reported laboratory demonstrations of bP IR photodetectors fabricated from single exfoliated bP flakes have demonstrated high room‐temperature specific detectivities of >10^10^ cm Hz^1/2^ W^−1^.^[^ ^8^, ^9^, ^22^ ^]^ These detectivity values can already compete with commercially available IR photodetector materials like HgCdTe and InGaAs. This performance is driven by bP's 2D layered crystal structure that has minimal surface dangling bonds resulting in extremely low surface recombination, with up to two orders of magnitude lower surface recombination velocity (SRV) than passivated silicon.^[^ ^23^ ^]^ The low SRV also allows bP films to be thinner than commercial MWIR materials, which in turn allows the reduction of volume‐dependent thermal noise, permitting improved signal‐to‐noise performance at room temperature. Furthermore, bP can easily form heterojunctions with other materials without needing to consider lattice matching, due to adaptable van der Waals bonding.^[^ ^9^, ^10^, ^14^, ^15^, ^17^ ^]^ bP also exhibits low bending rigidity and elastic deformation under moderate bending stress, which is suitable for incorporation into flexible devices,^[^ ^15^ ^]^ and bP is not composed of toxic heavy metals like commercial MWIR materials (e.g.,: Hg, Cd, As).

To date, most reported bP IR photodetectors are fabricated from single exfoliated flakes of bP with lateral sizes of <100 µm.^[^ ^3^, ^4^, ^5^, ^6^, ^8^, ^9^, ^10^, ^11^, ^12^, ^14^, ^15^, ^17^ ^]^ These single flake devices are useful for characterizing and benchmarking bP IR photodetectors compared to current technologies, however, maturation and commercialization requires scalable fabrication of the bP thin films over whole wafers/substrates for fabricating photodetector arrays. Bottom–up synthesis of bP thin films over whole wafers has proven to be difficult due to kinetic barriers that lead to preferential growth of the red phosphorous polymorph at ambient conditions,^[^ ^24^ ^]^ and preferential island growth of bP that lead to small grain sizes or amorphous films and low mobility of the synthesized bP films.^[^ ^25^, ^26^, ^27^ ^]^ Additionally, most reported synthesis methods require very high temperatures and pressure,^[^ ^26^, ^28^ ^]^ or are costly and complex.^[^ ^25^, ^27^, ^29^ ^]^ Instead, solution‐deposition methods may provide an avenue to create good quality scalable bP thin films with commercially viable throughput and cost. Some research teams have recently demonstrated preliminary electronic devices using solution‐deposited bP films, including some studies focusing on IR photodetection.^[^ ^7^, ^13^, ^30^, ^31^ ^]^ However, an approach is yet to be developed which allows the fabrication of high‐performance IR detector arrays at scale, due in part to unresolved issues related to liquid phase exfoliation (LPE) and film fabrication. The major issues with solution‐deposition methods are the damage caused to bP flakes during LPE of the bulk bP crystal and issues with the reproducibility of fabrication of scalable uniform thin films with high conductivity/mobility.^[^ ^32^, ^33^ ^]^

Typical LPE methods of 2D crystals such as ultrasonication or shear exfoliation apply inhomogeneous, randomized spikes of energy through the 2D crystal flakes that can result in excessive in‐plane tearing of flakes and limits the lateral size of the exfoliated flakes to nanometer dimensions, with size and thickness kept at a low, constant aspect ratio.^[^ ^34^ ^]^ However, larger lateral size, and higher aspect ratio flakes are preferred for lower resistance/higher mobility of solution‐deposited 2D crystal thin films.^[^ ^35^, ^36^ ^]^ Alternatively, electrochemical (EC) exfoliation is a controlled 2D crystal exfoliation method that can produce large lateral size flakes with high aspect ratio. EC exfoliation can obtain high aspect ratio flakes by intercalating ions (intercalants) between the 2D crystal layers.^[^ ^36^, ^37^, ^38^ ^]^ This increases inter‐flake distance, reducing the energy required for exfoliation, and applies the exfoliation energy directly in the 2D crystal interlayer, reducing in‐plane flake tearing.^[^ ^39^, ^40^ ^]^ The scalable film fabrication/printing method will also impact the bP thin film properties. Inkjet printing is a common method for scalable solution‐deposition of 2D nanomaterial thin films but the 2D crystal flake size in the ink is limited, due to the need to prevent clogging of the printing head, and many printing passes are required to build up sufficient film thickness for reasonable film conductivity and pinhole prevention.^[^ ^7^, ^41^, ^42^, ^43^ ^]^ Drop casting, spin coating, and blade coating also have issues with forming homogenous thin films from large 2D crystal flake sizes due to stringent ink rheology requirements.^[^ ^36^ ^]^ On the other hand, the vacuum filtration thin film fabrication method can produce scalable thin films of nanomaterials with controllable thickness/homogeneity and high mobility.^[^ ^44^, ^45^, ^46^, ^47^, ^48^ ^]^ Film thickness can be controlled by the volume of filtered nanomaterial dispersion with fixed concentration. In addition, this method also promotes homogenous coverage with minimal pinholes due to the constricted flow of the bP solution through the filter paper. That is, areas of the filter paper with more bP flakes deposited will restrict solvent flow through the filter pores, resulting in bP flakes flowing toward the less deposited areas of the filter paper. However, there is a scarcity of research reporting vacuum filtration fabrication of bP thin films, likely due to the difficulty in handling oxygen‐sensitive bP flakes.^[^ ^30^, ^49^, ^50^ ^]^

Here, we fabricate scalable thin films of bP through a tailored solution‐deposition method to fabricate high‐performance bP IR photodetector arrays. Bulk bP crystals are EC‐exfoliated and an additive‐free bP dispersion is prepared with a significant proportion of laterally large bP flakes (78% >2 µm) with minimal flake damage. The bP flake thickness is tuned to obtain thinner flakes for film uniformity and flexibility, but thick enough so that the bP bandgap remains bulk‐like to absorb IR light. bP thin films are prepared through the vacuum filtration method and transferred to device substrates as the semiconducting IR absorber layer for photoconductors. All steps of the fabrication method were performed in oxygen‐free environment with anhydrous solvents to minimize oxidation. Multiple IR photodetectors are then fabricated in arrays using scalable bP films on Au‐interdigitated electrodes (IDEs). Various capping layer materials that inhibit ambient degradation of the scalable bP films were tested for their compatibility with the bP thin films and their impact on the bP photodetector performance. Finally, the scalability of the bP thin film fabrication method was demonstrated by fabricating a linear array of 25 bP photodetectors and obtaining 25 × 25 pixel IR images. This work demonstrates a commercially viable, solution‐deposition method of fabricating scalable bP thin films for high‐performance optoelectronic devices including room temperature‐operable IR photodetector arrays.

## 2 Results and Discussion

**Figure 1:**
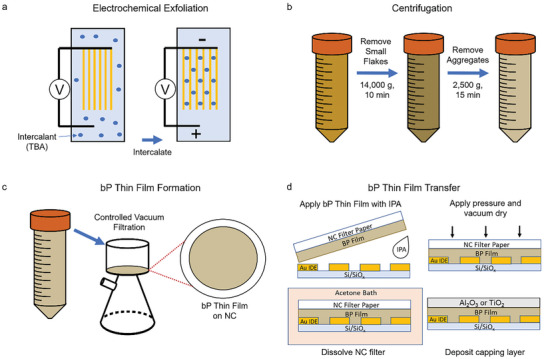
Schematic illustration showing the fabrication of bP thin films for photodetectors. a) EC‐exfoliation of bulk bP crystal into dispersible, laterally large bP flakes by intercalation of ions between the bP layers that reduce interlayer adhesion. b) Centrifugation of bP dispersion to obtain optimal bP flake distribution. c) Controlled vacuum filtration of EC‐exfoliated bP to form a bP thin film. d) Transfer of bP thin film to fabricate bP photodetectors.

Fabrication of the bP thin film IR photodetectors begins with controlled EC‐exfoliation of bulk bP crystal (**Figure** **1**a), followed by multiple centrifugation steps to obtain an additive‐free bP dispersion in dimethyl sulfoxide (DMSO) with large lateral flake sizes and optimized flake thickness distribution (Figure 1b). A bP thin film is then formed through controlled vacuum filtration of the bP dispersion onto nitrocellulose (NC) filter paper (Figure 1c). The bP thin film thickness can be controlled by varying the volume of bP dispersion filtered at a fixed bP dispersion concentration. The bP film is then transferred by inverting the filter paper and placing it onto the photodetector substrate with a thin layer of IPA solvent (Figure 1d). Drying the IPA under vacuum while applying pressure will adhere the bP film onto the substrate through the surface tension of the liquid bridge. The filter paper can then be dissolved through sequential acetone baths leaving the bP thin film transferred on the substrate. All previous steps were performed in a glovebox, with anhydrous solvents to prevent excess oxidation of the bP flakes. A capping layer is then deposited on the bP thin film to prevent ambient degradation of the bP thin film. This method can scalably and efficiently fabricate arrays of bP photodetectors over whole substrates for use in IR photodetector applications.

### 2.1 Electrochemical Exfoliation of bP and Incorporation into Thin Films

**Figure 2:**
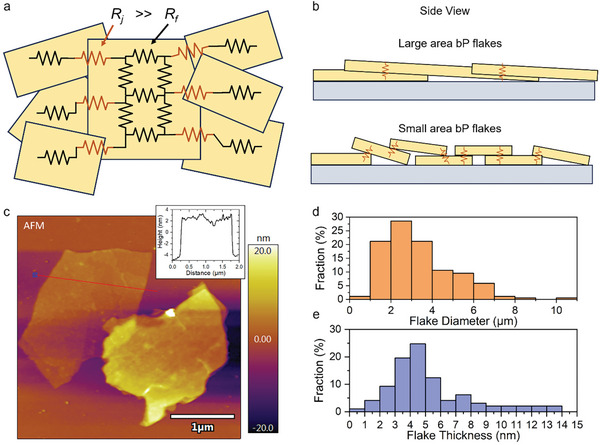
a) Electrical diagram of conductive nanosheet network that is formed from solution‐deposited bP thin films. Inter‐flake junction resistance (*Rj*) is much higher than intra‐flake resistance (*R_f_*) b) bP film with laterally larger bP flakes results in less, high‐resistance, inter‐flake junctions. c) Topographical AFM image of thicker EC‐exfoliated bP flakes with ≈6 and ≈15 nm thickness. Inset is a line profile marked by the red line in the main image showing the ≈6 nm thickness bP flake. d) Diameter distribution of *N* = 190 sample of bP flakes after EC‐exfoliation measured from TEM images. e) Thickness distribution of *N* = 97 sample of bP flakes after EC‐exfoliation and after centrifugation steps measured from AFM images.

The properties of the bP thin film are strongly dependent on the exfoliated bP flake properties. The resistivity of 2D nanomaterial films can be thought of as a complicated resistive network that comprises the resistivity and dimensions of the individual 2D flakes, and the number/magnitude of potential barriers at inter‐flake junctions as well as the flake overlap area, with many parallel pathways throughout the thickness of the thin film network (**Figure** **2**a).^[^ ^35^, ^36^ ^]^ However, this resistivity is most often dominated by the higher resistance inter‐flake junctions,^[^ ^35^ ^]^ so large lateral area flakes that reduce the inter‐flake junctions per film length can reduce the film resistivity (Figure 2b). Larger‐area flakes also increase the lower resistance intra‐flake parallel connections and increase the inter‐flake contact area, both reducing film resistance. Additionally, lower oxide thickness on the bP flake surfaces and fewer impurities trapped between the bP flake contacts will reduce the inter‐flake junction resistance.^[^ ^51^ ^]^ Accordingly, we aimed to produce large‐area bP flakes with minimal oxidation and no dispersion additives to improve the bP thin film resistivity.

Electrochemical exfoliation is an effective approach to precisely control the interlayer force applied to bulk bP crystals during exfoliation to reduce damage and in‐plane tearing of bP layers. During EC‐exfoliation, current is applied through the bulk bP in the presence of tetrabutylammonium (TBA) cations, causing the TBA to be inserted between (intercalating) the bulk BP crystal layers. Bulk bP typically has a closely packed layered structure, but after complete intercalation of TBA, a dramatic increase in the interlayer gaps occurs, expanding the bulk bP crystal and weakening the interlayer van der Waals interactions. This enables gentle exfoliation of laterally large bP flakes with high crystallinity after optimization of the EC exfoliation conditions. Figure 2c shows a topographical atomic force microscopy (AFM) image of bP flakes obtained from our optimized EC‐exfoliation method with lateral diameters of ≈2 µm and thicknesses of ≈6 and ≈15 nm. Figure 2d shows a sample distribution of bP flake lateral diameters (190 measured flakes, *N* = 190) after EC‐exfoliation obtained from TEM images. The majority (78%) of measured EC‐exfoliated bP flakes had >2 µm lateral diameter with a max. ≈10 µm diameter. These EC‐exfoliated bP flakes are large for LPE methods with a >1000 diameter/thickness aspect ratio compared to the typical 10–50 aspect ratio and <100 nm diameter obtained by ultrasonication,^[^ ^34^, ^52^ ^]^ or ≈100 aspect ratio obtained by microwave‐assisted exfoliation.^[^ ^32^, ^48^ ^]^ This consequently reduces inter‐flake junctions and resistivity of the bP thin films. This flake size is also large enough to span the µm‐scale IDE gaps used in the bP photodetectors demonstrated later in this study, further minimizing the role of inter‐flake junctions. Controlling the bP flake thickness is also important for improving the bP thin film properties. Thinner bP flakes will increase flake flexibility to ensure conformal contact between flakes, reduce step edge height to form a more homogenous thin film and reduce thermal noise. However, thinner bP flakes (i.e., less than 3 nm) exhibit a broader bandgap, reducing the spectral response of the bP flakes in the IR region.^[^ ^19^, ^20^, ^21^ ^]^ Hence, bP flakes with >3 nm thickness, yet <50 nm for bP film homogeneity and noise reduction, is therefore preferred for the fabrication of IR photodetectors. Approximatly 20% of the bP flakes were >3 nm thick after EC exfoliation as measured by AFM (Section S1, Supporting Information). The flake thickness distribution was then further controlled by multiple centrifugation steps, including removing supernatant after high‐speed centrifugation to reduce the <3 nm thick flake proportion, and removing sediment after low‐speed centrifugation to reduce the >50 nm thick flake and aggregate proportions. Figure 2e shows a sample distribution of bP flake thicknesses (97 measured flakes, N = 97) after EC‐exfoliation and the centrifugation steps obtained from AFM measurements. The proportion of bP flakes >3 nm thick became >85% after the centrifugation steps with a mean flake thickness of 6.2 nm.

**Figure 3:**
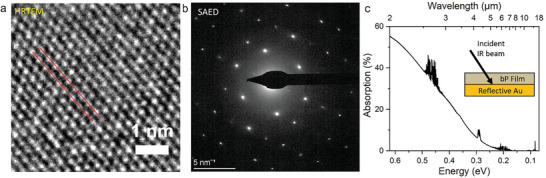
a) HRTEM image and b) SAED pattern of EC‐exfoliated bP showing ≈0.19 nm lattice spacing and single crystal structure. c) Absorption spectrum obtained from transflection‐mode FTIR spectroscopy of 120 nm mean thickness bP thin film on Au used for bP photodetectors. The inset diagram shows the planar sample structure used for the measurement.

**Figure** **3**a,b illustrate the high‐resolution transmission microscopy (HRTEM) image and selected‐area electron diffraction (SAED) pattern of the EC‐exfoliated bP flakes. As shown in Figure 3a, the lattice spacing of the bP was measured to be 0.19 nm, which is consistent with the reported values in the literature.^[^ ^53^, ^54^ ^]^ Moreover, the SAED pattern depicted in Figure 3b suggests a single crystal structure of bP. Absorption spectra of the fabricated bP thin films were obtained from transflection‐mode Fourier‐transform infrared spectroscopy (FTIR) spectroscopy measurements and presented in Figure 3c to determine suitability for IR photodetection after processing. Transflection‐mode FTIR spectroscopy measures the sum of transmission and reflection of the thin film on a reference reflective surface (Au) (Figure 3c **inset**), where the absorption of the thin film stack is equivalent to 1‐transflection. Infrared IR absorption up to the MWIR region can be observed from the absorption spectrum, which is expected from the bulk bP bandgap of ≈0.3 eV. A defined optical bandgap could not be fitted from the Tauc plot as the films consist of a distribution of bP flake thicknesses, each with different bandgaps, which superimpose to form the absorption spectrum.^[^ ^55^ ^]^

### 2.2 bP Thin Film Oxidation and Capping Layers

**Figure 4:**
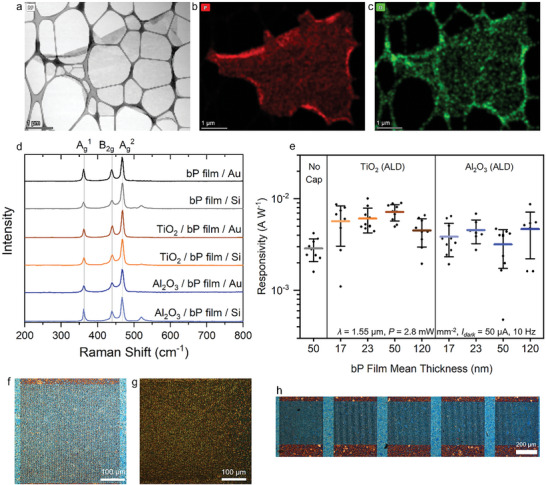
a) Bright‐field HAADF image of a ≈3 µm diameter EC‐exfoliated bP flake and the correlated b) phosphorus and c) oxygen EDX elemental maps taken simultaneously, showing some slight oxidation of bP flake surface after air exposure. d) Raman spectra of bP thin films in fabricated bP photodetectors. e) Responsivities of bP photodetectors with different capping layers and bP film thickness under λ = 1.55 µm illumination at room temperature (*P* = 2.8 mW mm^−2^, *I_dark_* = 50 µA, 10 Hz). Each data point is one photodetector and all photodetectors of the same type were fabricated on the same substrate from the same bP thin film. The middle line is average *R* and whisker ends are 1 standard deviation. f–h) Optical microscope images of bP thin films transferred on Au IDE for bP photodetectors. f) 50 nm mean thickness bP film, g) 120 mean thickness bP film, h) 50 nm mean thickness bP film over multiple IDEs forming a 5 × 1 bP photodetector array.

Black phosphorus tends to degrade under ambient conditions (oxygen, water, and light) and convert to phosphorous oxide (P_x_O_y_) that can be dissolved in ambient water from humidity, which eventually completely corrodes the bP.^[^ ^56^, ^57^, ^58^, ^59^ ^]^ To reduce oxide formation around each bP flake, the whole bP thin film fabrication process was performed in a glovebox with anhydrous solvents. This reduces oxide formation on the bP flakes in ambient conditions and reduces inter‐flake junction resistance.^[^ ^56^ ^]^ The bP dispersion was also thoroughly cleaned of intercalant and no additives were added to the dispersion, reducing inter‐flake resistance from contaminants. The effect of ambient conditions on bP can be seen by scanning transmission electron microscopy (STEM) images we obtained of the EC‐exfoliated bP flake after air exposure (during sample preparation), including a high‐angle annular dark‐field (HAADF) image in bright‐field mode (**Figure** **4**a) and correlated energy‐dispersive X‐ray spectroscopy (EDX) elemental maps of phosphorus (Figure 4b) and oxygen (Figure 4c). The oxygen EDX map reveals some surface oxidation of the bP flake after air exposure highlighting the importance of minimizing ambient exposure before bP thin film formation.

To protect the bP thin films after fabrication and during bP photodetector operation, capping layers were deposited on the fabricated bP thin films which prevent the ambient degradation and reduce recombination active trap states at bP flake edges/surfaces. Different capping layers were tested including physical vapor deposition (PVD) of Al_2_O_3_ and MoO_3_ and atomic layer deposition (ALD) of TiO_2_ and Al_2_O_3_.^[^ ^59^, ^60^, ^61^ ^]^ The results from these tests are provided in Supporting Information Section S2 showing that the ALD‐deposited capping layers exhibited the highest performance. The ALD‐deposited films likely show the best performance because they introduce less defects to the bP from kinetic damage compared to PVD‐deposited capping layers. Further, ALD is known to provide excellent conformal coatings, allowing coverage of the bP flakes providing good passivation and protection. TiO_2_ and Al_2_O_3_ capping layers were chosen specifically as they have >99% transmission over the SWIR/MWIR region and have been used previously for 2D nanomaterial IR photodetectors.^[^ ^62^, ^63^, ^64^ ^]^ As such, the ALD‐deposited TiO_2_ and Al_2_O_3_ capping layers were chosen as a focus in this study. The degree of oxidation in the bP thin films was then characterized by their Raman spectra.^[^ ^65^ ^]^ Figure 4d shows Raman spectra obtained for the bP thin film deposited on the Au IDEs, with Al_2_O_3_ and TiO_2_ capping layers, as well as no capping layer. For each case, spectra are obtained in a region above the Au IDEs and above the Si/SiO_2_ substrate. All samples exhibited Raman spectra peaks at ≈467.8, ≈439.9, and ≈362.2 cm^−1^, which can be assigned to the bP Raman modes of A_g_^2^, B_2g_, and A_g_1, respectively.^[^ ^66^, ^67^ ^]^ A_g_1/A_g_^2^ Raman mode peak integral area ratios were measured at 0.30 ± 0.08 which indicates that the fabrication method introduces only minimal oxidation to the bP thin film (A_g_1/A_g_^2^ > 0.2 for low oxidation levels ^[^ ^65^ ^]^). The difference between A_g_^2^ and B_2g_ peak positions is ≈27.9 cm^−1^ which is similar to literature values obtained for mechanically exfoliated bulk bP flakes on Si/SiO_2_ substrates.^[^ ^67^ ^]^ The sharp Raman spectra peaks correlating with known bulk bP Raman modes indicate the bP flakes in the thin film maintain high crystallinity and bulk‐like properties after EC‐exfoliation and thin film fabrication. Minimal oxidation during fabrication is also supported by X‐ray photoelectron spectra of the EC‐exfoliated bP before air exposure, provided in Supporting Information Section S1, which show a relatively low level of oxidation of the bP flakes compared to previously reported solution‐exfoliated bP flakes/films.^[^ ^68^, ^69^, ^70^ ^]^ MWIR photoluminescence (PL) of the bP thin films was then measured to confirm the optoelectronic quality of the bP thin films using a λ = 660 nm excitation laser, with bP's PL emission detected after a 2400 nm longpass filter (details in Section S3, Supporting Information). The measured PL quantum yields (PLQYs) of all three films were very similar with values varying between 0.15–0.17%, which is comparable to previously reported freshly exfoliated, 4–10 nm thick, bP flake PLQY.^[^ ^16^, ^23^ ^]^

Next, the influence of bP film thickness and ALD‐deposited capping layer on the bP photodetector performance is investigated. Responsivities (*R*) are first measured under λ = 1.55 µm illumination for 10‐device ALD‐capped bP photodetectors arrays with bP film thicknesses of 17, 23, 50, and 120 nm (Figure 4e). Note that the bP film thickness refers to the aggregate bP film formed on the IDEs which are composed of many bP flakes deposited together as an aggregate film with a mean individual flake thickness of 6.2 nm. The mean and standard deviation of the measured *R* in each array are presented in **Table** **1** for clarity. The film thickness is controlled by varying the volume of the filtered bP dispersion at a fixed bP concentration. The measurement conditions were standardized by using a source drain bias (*V_DS_*) value for each thickness which achieves constant *I_dark_* = 50 µA when not illuminated. Thicker bP film photodetectors required lower *V*_DS_ to achieve a given *I_dark_* due to lower bP film resistance (*V_DS_* = ≈1.9, ≈0.95, and ≈0.6 V required for bP film thickness of 17, 50, and 120 nm, respectively), which is expected from the increasing cross‐sectional area of the conduction channel. While the responsivity (*R*) of ALD‐deposited TiO_2_ and Al_2_O_3_ capped devices was consistently higher than the uncapped devices, the effect of the bP film thickness on *R* was small. As such, the thicker 120 nm bP films were chosen as a focus for the remainder of the study as a lower bias was required to achieve a given *R*. Figures 4f–h provide optical microscope images of the fabricated bP photodetectors with (Figure 4f) thinner and (Figure 4g) thicker bP films, demonstrating the coverage of the bP film over the whole Au IDE area, and coverage over multiple Au IDEs (Figure 4h). The cross‐section of bP photodetectors with ≈120 nm thickness bP films were also imaged by high‐resolution helium ion microscopy (HIM) (Section S4, Supporting Information), and they show good adhesion of the bP films to the substrate.

**Table 1:** Responsivity mean and standard deviation for 10‐device bP photodetector arrays (λ = 1.55 µm illumination, room temperature, *P* = 2.8 mW mm^−2^, *I_dark_* = 50 µA, 10 Hz) (Plot in Figure 4e).

Capping Layer	None	TiO_2_ (ALD)				Al_2_O_3_ (ALD)			
bP Film Mean Thickness (nm)	50	17	23	50	120	17	23	50	120
Array Responsivity Mean (mA W^−1^)	2.86	5.69	6.07	7.17	4.5	3.85	4.55	3.17	4.68
Array Responsivity Std. Dev. (mA W^−1^)	0.80	2.65	1.83	1.45	1.53	1.53	1.31	1.43	2.47

### 2.3 Optimized bP Film IR Photodetector Characterization

**Figure 5:**
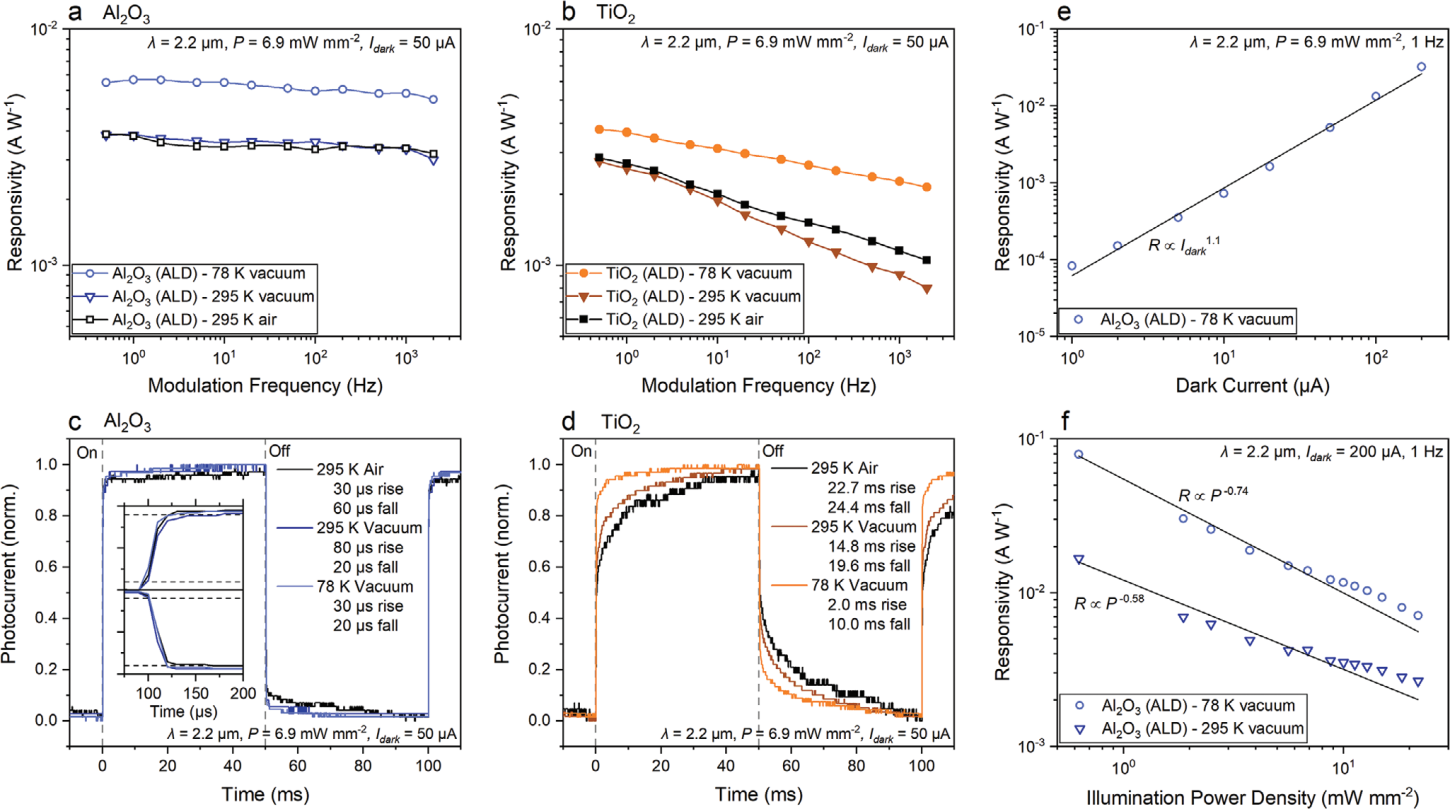
Characterization of bP photoconductors under λ = 2.2 µm illumination. Frequency response of a) Al_2_O_3_‐capped and b) TiO_2_‐capped bP photodetectors in different environments: room temperature/air (black squares), room temperature/vacuum (triangles), 78 K/vacuum (circles) (*I_dark_* = 50 µA, *P* = 6.9 mW mm^−2^). Photoresponse of c) Al_2_O_3_‐capped and d) TiO_2_‐capped bP photodetectors to square wave‐modulated IR illumination in different environments (*I_dark_* = 50 µA, *P* = 6.9 mW mm^−2^, 10 Hz, inset 100 Hz). e) *R* dependence on *I_dark_* for Al_2_O_3_‐capped bP photodetector (1 Hz, *P* = 6.9  mW mm^−2^) f) *R* dependence on *P* for Al_2_O_3_‐capped bP photodetector in 78 K/vacuum (circles) and room temperature/vacuum (triangles) (*I_dark_* = 200 µA, 1 Hz).

The optimized bP photodetectors were then characterized in detail to quantify their sensitivity and speed figures of merit. First, **Figure** **5**a,b provide the frequency‐dependent *R*, measured using λ = 2.2 µm illumination, for the Al_2_O_3_‐capped and TiO_2_‐capped photodetectors, respectively. The Al_2_O_3_‐capped bP photodetectors exhibit slightly higher *R* with weaker frequency dependence compared to the TiO_2_‐capped bP photodetectors. This indicates that TiO_2_‐capped bP photodetectors may have more trap states introduced at the TiO_2_/bP interface that contribute to the slower response.^[^ ^71^ ^]^ The *R* of both TiO_2_‐ and Al_2_O_3_‐capped devices improved at low temperatures (78 K), likely due to improved collection efficiency and higher mobility.^[^ ^72^ ^]^ The time‐dependent photocurrent of Al_2_O_3_‐ and TiO_2_‐capped bP photodetectors under square‐wave modulated illumination was then measured as shown in Figure 5c,d. The Al_2_O_3_‐capped bP photodetector exhibited fast rise/fall times of 30/60 µs, which were found to be independent of temperature. These µs‐scale response times are over an order of magnitude faster than those previously reported for scalable solution‐deposited bP photoconductors,^[^ ^7^, ^30^ ^]^ and some earlier single‐flake bP photoconductors/phototransistors.^[^ ^3^, ^6^ ^]^ The bP films have minimized defects due to the low oxidation and low damage fabrication process employed, and are well passivated from the Al_2_O_3_ capping layer, which may generate fewer/shallow trap states that allow response speeds closer to well‐fabricated single flake bP photoconductors. The µs rise/fall times suggest the photoconductive effect is the dominant photocurrent‐generating mechanism in these devices, rather than the ms regime rise/fall times typical of photobolometers. Additionally, there was no observable photocurrent under zero bias and the contacts and illumination were symmetrical and uniform, suggesting the photothermoelectric effect is not a significant contributor. The TiO_2_‐capped bP photodetector had slower room temperature rise/fall times of 23/24 ms, which improved to 2.0/10 ms at low temperatures, which aligns with the frequency‐dependent measurements in Figure 5b (discussion in Section S5, Supporting Information). Based on the higher *R* and faster response time, the Al_2_O_3_‐capped bP photodetectors exhibit superior performance and hence are the focus of the remaining study.

The *R* of the Al_2_O_3_‐capped bP photodetector is dependent on the bias conditions and the illumination power density (*P*). Figure 5e shows the increase in *R* with increased *V*_DS_, represented as increasing *I_dark_* in the plot. The *R* shows a slightly superlinear dependence on *I_dark_* across the entire range of bias conditions (i.e., *R* ∝ *I_dark_* ^1.1^), with the highest *R* values obtained under the largest bias, as is expected for photoconductors. Figure 5f provides the *R* dependence on *P* for the Al_2_O_3_‐capped bP photodetector. The *R* has an inverse, non‐linear *P* dependence (*R* ∝ *P* ^−0.58^ at room temperature and *R* ∝ *P* ^−0.74^ at 78 K), with champion values of *R* = 16.7 and 79.6 mA W^−1^ obtained at low *P*, for room temperature and 78 K, respectively. This behavior is indicative of the gradual saturation of trap states in the device with increasing *P*, which is commonly observed in 2D material photoconductors.^[^ ^73^ ^]^ From these *R* values and their corresponding *I_dark_* values, a specific detectivity (D*) can be calculated for a photoconductor from estimated shot noise by D* = *R* (*A*/2*qI_dark_*)^1/2^, where *A* is the photodetector area and *q* is the elementary charge. Based on this, the Al_2_O_3_‐capped bP photodetector yielded a peak D* of 4.0 × 10^8^ cm Hz^1/2^ W^−1^ at 78 K, and 8.3 × 10^7^ cm Hz^1/2^ W^−1^ at room temperature, under λ = 2.2 µm illumination. We emphasize that this is an upper limit estimate of *D* ^*^ as 1/*f* (flicker) noise can be significant for photoconductive devices at low frequency resulting in an overestimation of *D* ^*^.^[^ ^74^, ^75^ ^]^ In addition, internal gain in photoconductive devices can also increase noise,^[^ ^74^, ^76^ ^]^ but we do not believe this to be a significant factor in this study (Section S5, Supporting Information). The stability of the Al_2_O_3_‐capped bP photodetector was characterized by measuring the photodetector *R* ≈5 months after initial fabrication, with only a ≈4.1% decrease of *R*. During this 5‐month period, samples were subjected to multiple measurement cycles in air separated by storage in a crude vacuum. The above responsivities are greater than other solution‐deposited bP thin film photodetectors measured at similar wavelengths and *V_DS_* without external gating,^[^ ^7^, ^13^, ^30^, ^31^ ^]^ and the above responsivities/detectivities are also approaching individual‐flake bP photodetectors measured at MWIR wavelengths,^[^ ^4^, ^5^, ^8^, ^9^, ^11^, ^77^ ^]^ demonstrating the feasibility of the technique reported in this work for scalable fabrication of bP optoelectronic devices.

The EC exfoliation method and the film fabrication in a low‐oxygen environment enabled the scalable fabrication of high‐quality bP thin films and photodetector arrays. These methods produce bP films that contain bP flakes with larger areas compared to previous reports and low damage/oxidation. Exfoliation of bP flakes of this size is difficult using other solution‐based methods and vacuum filtration has rarely been reported for fabricating bP films due to the oxidation of the bP during film formation and transfer to substrate.

It should be noted that the thin film bP photoconductors in this work have a simple architecture, which was chosen to demonstrate the inherent quality of the fabricated bP thin films. Adding gate modulation to these devices could improve *R* by reducing hole transport barriers and increasing the channel hole concentration.^[^ ^5^, ^73^ ^]^ For a back‐gated photoconductor, this would require deposition of the Au IDEs on top of the bP thin film to obtain effective gate modulation, as having these contacts underneath the bP photoconductor results in poor gate modulation (as shown in Section S6, Supporting Information). In addition, employing light trapping strategies such as anti‐reflection layers, optical cavities,^[^ ^14^ ^]^ or using the absorber itself as a waveguide,^[^ ^9^ ^]^ would also improve the overall absorption of the bP thin film and hence *R* of the devices. The same bP thin film fabrication process could be also used with black phosphorus arsenic alloys to extend the spectral response further into the MWIR region.^[^ ^8^ ^]^

### 2.4 bP Film Photodetector Array IR Camera

**Figure 6:**
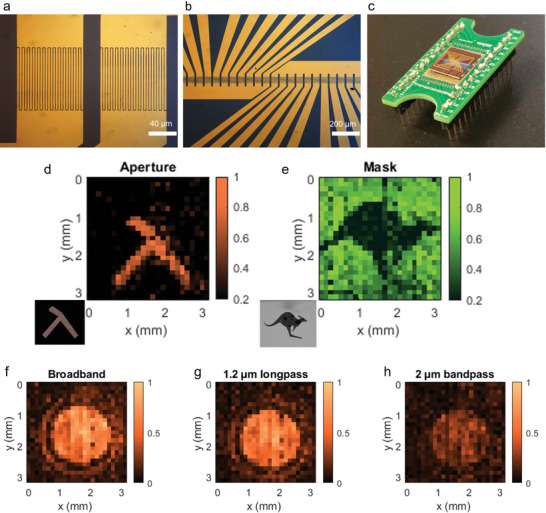
a, b) Microscope images of the bare 25‐pixel linear array of Au IDEs on Si/SiO_2_ wafer used for fabricating the 25‐pixel bP IR camera. c) Image of the bP photodetector linear array mounted on the 28‐pin chip carrier. Normalized 25 × 25 pixel IR images of λ = 1.5 µm illumination through a d) “λ”‐shape aperture and a, e) kangaroo‐shape mask with ≈203 ppi resolution using the bP IR camera (insets are microscope images of aperture and mask). f) Normalized 25 × 25 pixel IR images of 1500 K blackbody broadband illumination using the bP IR camera, and the same images through g) a λ = 1.2 µm longpass filter and h) a λ = 2 µm bandpass filter with 500 nm full‐width at half maximum.

To demonstrate the scalability of this approach, 25 Al_2_O_3_‐capped bP photodetector pixels were fabricated on a cm‐scale chip to make a simple linear array camera. These bP photodetector pixels were fabricated, using an identical method to that described previously, onto 100 × 100 µm IDEs with 2 µm gaps (**Figure** **6**a). Individual pixels are separated by a 130 µm pitch in a 25‐pixel linear array (Figure 6b). The bP film is applied over the entire pixel array, capped with a ≈15 nm Al_2_O_3_ layer, after which the chip is mounted into a 28‐pin chip carrier and connected to read‐out circuitry (Figure 6c). The chip carrier is mounted onto a motorized stage, which during a measurement, steps down in increments of ≈130 µm to obtain 25 × 25 pixel images. Figure 6d,e shows images captured by the bP IR camera when λ = 1.5 µm illumination is directed through a “λ”‐shape aperture and kangaroo‐shape mask. This wavelength illumination was selected because the light source was able to produce a very uniform power density over a large area (3.1 × 3.1 mm) which allowed for a more accurate representation of the bP photodetector array uniformity. As shown in Figure 6f, the bP IR camera could also capture images of 1500 K blackbody broadband IR illumination focused onto the IR camera active area, demonstrating its response to broadband light. We also applied λ = 1.2 µm longpass filter (Figure 6g) and a λ = 2 µm bandpass filter with a 500 nm full‐width at half maximum (Figure 6h) to demonstrate the response of the bP IR camera to only IR illumination. The bP IR camera photocurrent reduces only slightly when the λ = 1.2 µm longpass filter is used, indicating that the majority of the spectral response of the bP IR camera is in the λ > 1.2 µm IR region. These images show that the fabricated bP IR camera can obtain high spatial resolution at IR wavelengths and serve as a preliminary demonstration of the scalability of the EC‐exfoliated, vacuum filtration‐deposited bP film devices.

IR imaging systems composed of bP photodetector arrays could provide distinct advantages over conventional HgCdTe or InGaAs photodetector arrays. As mentioned in the introduction, bP flakes can have low SRV and hence could be used to fabricate thinner devices with the potential for lower noise. The bP photodetectors may also be incorporated into flexible or curved devices. Finally, HgCdTe and InGaAs films are fabricated through complex and expensive epitaxial growth methods, so using these solution‐deposited bP thin films could enable much cheaper and simpler fabrication of IR photodetector arrays. Further increases to D^*^ are still required to compete with commercial materials, however, switching to a photodiode architecture, incorporating light‐trapping strategies, and further suppression of defect formation could all provide improvements to D^*^. While the use of bP as an IR photodetector material is still in its infancy, this work provides a step toward the realization of a commercially relevant fabrication pathway for such devices.

## 3 Conclusion

In this study, a scalable solution‐deposition method for large area and uniform bP thin films is developed and used to fabricate IR photodetector arrays. The high‐quality bP thin films were composed of laterally large and thickness‐optimized bP flakes with minimal oxidation, which enabled absorption into the MWIR spectral region corresponding to bP's bulk bandgap. The EC exfoliation and low‐oxygen film fabrication methods reported enabled the scalable fabrication of the high‐quality bP thin film and photodetector arrays. After optimizing bP film thickness and integrating an Al_2_O_3_ capping layer, bP IR photodetectors are fabricated achieving *D* ^*^ up to 4.0 × 10^8^ cm Hz^1/2^ W^−1^ with fast 30/60 µs rise/fall times, under λ = 2.2 µm illumination. The *R*, D*, and rise/fall times represent a significant improvement on the few previously reported solution‐deposited bP thin film photodetectors. Finally, we demonstrate the scalability of the bP thin film fabrication technique by constructing a linear array of 25 bP photodetectors and obtaining 25 × 25 pixel IR images at ≈203 ppi. This research demonstrates a commercially viable method of fabricating scalable bP thin films for high‐performance optoelectronic devices, including room temperature‐operable IR photodetector arrays.

## 4 Experimental Section

### Materials

Chemicals used as received were dimethylsulfoxide (DMSO) (analytical grade (≥99.9%), Fisher Scientific), N‐methyl‐2‐pyrrolidone (NMP) (analytical grade (≥99.9%), Sigma–Aldrich), anhydrous isopropanol (IPA) (95%, Sigma–Aldrich), anhydrous acetone (95%, Sigma–Aldrich), tetrabutylammonium (TBA) hydrogensulfate (Sigma–Aldrich), molybdenum trioxide powder (MoO_3_), titanium isopropoxide (TTIP) and dimethylaluminum isopropoxide (DMAI). Bulk BP crystal (99.998% pure) was obtained from Smart Elements.

### bP Electrochemical Exfoliation

The electrochemical expansion of bP was carried out in a two‐electrode packed‐bed electrochemical reactor (PBER) under a constant current of −1 mA. Briefly, 20 mg bulk bP was pressed against a conductive boron‐doped diamond (BDD) (Diaccon GmBH) working electrode and separated from a platinum (Pt) counter electrode (Pt wire, length 33 cm, 0.25 mm diameter, purity 99.99%; Goodfellow) using a glass fiber membrane (GF/B; Whatman). A 0.5 kg weighted press was placed on top of the glass fiber membrane to ensure the bP bed made intimate contact with the BDD. A 5 mL nonaqueous electrolyte of 0.1 m TBA hydrogensulfate in DMSO was pipetted into the reactor. A Gamry electrochemical workstation (Interface 1010E) was employed for the galvanostatic charging (GC) with the limiting voltage set at −8.0 V. After complete intercalation, the expanded bP was further exfoliated and dispersed in 10 mL DMSO via bath sonication for 5 min. The bP dispersion was then centrifuged (14 000 g, 10 min) and the supernatant was extracted and disposed of. The bP solid was then redispersed in fresh 10 mL DMSO, repeating three times in total to remove excess intercalant and very small/thin bP flakes. The bP dispersion was then centrifuged at a lower speed (2500 g, 15 min) and the supernatant was collected as the final bP dispersion, to remove large aggregates of bP.

### bP Thin Film and bP Photodetector Fabrication

Interdigitated electrodes were deposited on the Si/SiO_2_ substrates using a photolithography/liftoff process, with electron‐beam evaporation used to deposit the 10 nm Cr/30 nm Au layers. The IDEs in Figures 4 and 5 had 400 × 400 µm active area, 2 µm width gaps, 2 µm width fingers. The IDEs in Figure 6 had 100 × 100 µm active area, 2 µm width gaps, and 2 µm width fingers.

To form bP thin films, 0.1–1 mL of EC‐exfoliated bP dispersion was added to 40 mL of anhydrous IPA and then immediately vacuum filtered on nitrocellulose filter paper (0.45 µm pore size). The bP thin films were then transferred by placing a few drops of anhydrous IPA on the IDE substrates, pressing the bP thin film against the IDEs, and then vacuum drying (room temperature, 30 min) while clamped together with Teflon sheets. The NC filter paper was then dissolved in three sequential anhydrous acetone baths (30 min each). All processes were performed in the N_2_ glovebox except centrifuging and vacuum drying during bP film transfer.

Finally, capping layers were deposited on the bP thin films. Atomic layer deposition was used to deposit ≈13 nm of TiO_2_ and ≈13 nm of Al_2_O_3_ using TTIP and DMAI, respectively, both at 90 °C (Figures 4, 5, 6). For photodetector characterization and IR camera operation, the bP photodetector substrates were mounted onto 28‐pin chip carriers with wire‐bonded connections to each side of the IDEs.

### bP Flake and bP Thin Film Characterization

Atomic force microscopy (AFM) was performed in the air using Asylum Research Cypher S with Asylum Research software, operating in standard tapping mode configuration using an AIR cantilever holder. TEM measurements were performed using a HITACHI HT7700‐A EDS for the statistical analysis of the size and area distribution, and a FEI Titan Themis for the selected area electron diffraction (SAED) images. Scanning transmission electron microscopy (STEM) imaging and Energy‐Dispersive X‐ray (EDX) elemental mapping were carried out also on a FEI Titan Themis S‐TEM instrument. The STEM probe was aberration‐corrected, enabling sub‐angstrom spatial resolution, and HAADF images were obtained. Raman spectra were obtained from a confocal Raman microscope (Renishaw InVia Qontor) using a 532 nm excitation laser, 1800 cm^−1^ grating, and a 50× long working distance objective. Each measurement was averaged over ten accumulations with 1 s integration time. Absorption spectra were obtained from an FTIR spectrometer (Frontier, Perkin Elmer) in transflection mode using a bare, clean Au surface as the reference for 100% transflection.

### bP IR Photodetector Characterization

The individual bP photodetector figure‐of‐merit characterization was performed using two electrically modulated IR laser diodes which emit λ = 1.55 µm (Figure 4e) and λ = 2.2 µm (Figure 5) light. Source drain biases were applied using a current preamplifier (SR570, Stanford Research Systems) to achieve a specific *I_dark_* value (for example 50 µA or 200 µA). Photoresponse behavior was recorded using an oscilloscope or lock‐in amplifier (SRS 860, Stanford Research Systems). The power density at the detector surface from the λ = 1.55 µm laser diode was measured using a calibrated Ge detector at the same position/distance as characterized photodetectors behind a 400 µm circular aperture. Similarly, power density from the λ = 2.2 µm laser was calibrated using a broadband thermal sensor (S401C, Thorlabs) and power meter (PM100D, Thorlabs) at the same position/distance as characterized photodetectors again behind a 400 µm circular aperture.

### bP IR Camera Fabrication and Operation

The bP IR camera (Figure 6) was connected to a multiplexer and micromotor‐controlled stage for operation. To obtain images, each pixel was selected in sequence by the multiplexer and a bias was applied to obtain a constant *I_dark_* = 10 µA for each pixel. The light source was mechanically chopped at 80 Hz and photocurrent at each pixel was obtained using a lock‐in amplifier (SRS 860, Stanford Research Systems). After obtaining photocurrent from each pixel of the array, the bP IR camera was stepped down by 130 µm to capture another line of the array, until a 25 × 25 pixel image was obtained. A λ = 1.55 µm laser diode coupled to a single mode fiber (FPL1009S‐1550 nm) at a long working distance (≈4 cm) was used to obtain a uniform illumination power density for capturing the IR images (Figures 6d,e). The aperture and mask were placed close to the bP IR camera for the aperture/mask IR images. Each pixel in the images (each column) was normalized by the pixel's *R* as determined by averaging the photocurrent obtained for each pixel from the uniform illumination of the λ = 1.55 µm source (Section S7, Supporting Information). A 1500 K blackbody broadband IR illumination source (SLS203L/M, Thorlabs) was used for the spectral images (Figures 6f–h). A 1.2 µm longpass filter or 2.0 µm bandpass filter (500 nm full‐width at half maximum) was placed in the beam path for the spectral images and normalized (Section S8, Supporting Information).

## Conflict of Interest

The authors declare no conflict of interest.

## Supporting information

Supporting Information

## Data Availability

The data that support the findings of this study are available from the corresponding author upon reasonable request.
