# Supplementary material for: Scalable Fabrication of Black Phosphorous Films for Infrared Photodetector Arrays
Source: Adv Sci (Weinh). 2024 Jul 21;11(35):2403182. doi: 10.1002/advs.202403182 (PMC11575499; doi:10.1002/advs.202403182)
Supplement: Supplementary file 1 — Supporting Information [file ADVS-11-2403182-s001.pdf]

Supporting Information

Scalable Fabrication of Black Phosphorous Films for Infrared Photodetector Arrays

Alexander Corletto,[1] Purevlkham Myagmarsereejid,[2] Shifan Wang,[1] Wei Yan,[1] Sivacarendran Balendhran,[1] Huan Liu,[3] Yu Lin Zhong,[2] Kenneth B. Crozier,[1,3,4] Munkhbayar Batmunkh,[2] and James Bullock[1*]*

[1] Department of Electrical and Electronic Engineering, The University of Melbourne, Parkville, Melbourne, Victoria 3010, Australia

[2] Queensland Micro- and Nanotechnology Centre, School of Environment and Science, Griffith University, Nathan campus, Brisbane, Queensland 4111, Australia

[3] School of Physics, The University of Melbourne, Parkville, Melbourne, Victoria 3010, Australia

[4] ARC Centre of Excellence for Transformative Meta-Optical Systems, The University of Melbourne, Parkville, Melbourne, Victoria 3010, Australia

[*] Corresponding Authors: james.bullock@unimelb.edu.au, m.batmunkh@griffith.edu.au

Supporting Information 1 – Additional EC-bP Flake Characterization

The thickness distribution of EC-exfoliated bP flakes was determined by atomic force microscopy (AFM) of the individual exfoliated flakes (**Figure S1a**). EC-exfoliated bP flake dispersion was drop-cast on clean, low roughness Si/SiO_2 substrate and the solvent evaporated under vacuum. Topographical images of the bP flakes on substrate were obtained in air tapping mode and the bP flake thickness was measured by line profiles of the edges of bP flakes in the AFM images. To compliment the Raman spectroscopy data, we have also obtained a high-resolution phosphorus-region X-ray photoelectron spectroscopy (XPS) spectrum of an individual bP flake after the electrochemical exfoliation process to monitor the level of oxidation of the bP flakes that are placed into the bP aggregate film of the bP photodetectors (**Figure S1b**). The peak at ~ 132.9 eV binding energy, which is assigned to oxidized phosphorus (P-O), is much smaller than the peaks at ~129.3 and ~130.1 eV, which are assigned to phosphorus-bonded phosphorus (P-P) $2p_{3/2}$ and $2p_{1/2}$, respectively. This demonstrates the low level of bP flake surface oxidation caused by the electrochemical exfoliation process compared to previously reported solution-exfoliated bP flakes/films.[1-3]

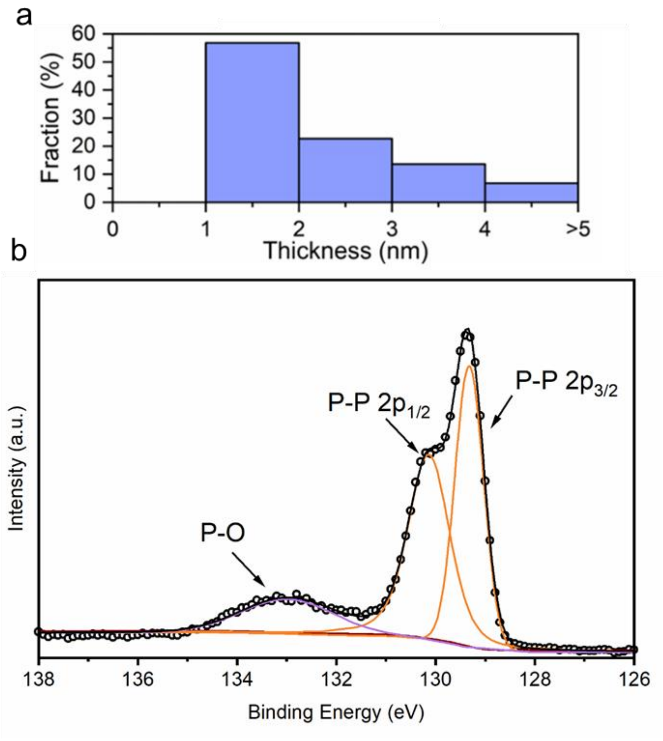

Figure S1. a) Thickness distribution of N=50 sample of bP flakes after EC-exfoliation but before centrifugation steps measured from AFM images. **b)** High-resolution phosphorus-region (P 2p) XPS spectrum of an individual bP flake after the EC-exfoliation process. Binding energy was calibrated by setting the C-C peak in C1s spectrum from adventitious carbon as 284.8 eV.

Supporting Information 2 – Other Capping Layer Materials for bP Photodetectors

Figure S2 provides the responsivities (*R*) obtained from bP photodetectors with different capping layers, including electron-beam evaporation-deposited Al_2O_3, thermal evaporation-deposited MoO_3, a combination of MoO_3 and Al_2O_3, and atomic layer deposition (ALD)-deposited TiO_2. MoO_3 deposited on bP FETs has been reported to increase hole mobility from p-type doping and result in a large increase of (*R*) of the bP FETs.[4] 10 nm MoO_3 was deposited by thermal evaporation at ~0.2 Å s^{-1} and 20 nm Al_2O_3 was deposited by electron-beam evaporation at 0.1 Å s^{-1} at < 10^{-6} Torr pressure. Additionally, as another method to prevent degradation of the bP thin films, 5 %v/v N-methyl-2-pyrrolidone (NMP) was added to a set of bP dispersions before the vacuum filtration step to form bP films. NMP has been reported to potentially reduce ambient degradation of bP after exfoliation in bP/NMP dispersions.[5] The responsivities were obtained using a λ = 1.55 µm laser at 2.8 mW mm^{-2} power density and 10 Hz pulse frequency. The ALD-deposited TiO_2 exhibited the highest average *R*, although only marginally more than uncapped devices. Devices with electron-beam evaporation-deposited Al_2O_3 capping layers demonstrated negligible difference in *R* to the uncapped devices, which may be due to the high kinetic energy of depositing Al_2O_3 particles introducing defects in the bP film that counter any improvement to *R*. Additionally, MoO_3 capping layers exhibit a slight decrease in *R*. The addition of NMP to the bP dispersion before bP film fabrication yielded mixed and small changes to *R*, with NMP marginally increasing average *R* of the no capping layer and TiO_2 capping layer photodetectors. From these results we deduced that ALD-deposited metal oxides would be the most effective capping layers and that the addition of NMP to the bP film formation step is not worth the negligible improvement. The IDEs in **Figure S2** had 800 µm × 800 µm active area, 5 µm width gaps, 5 µm width fingers.

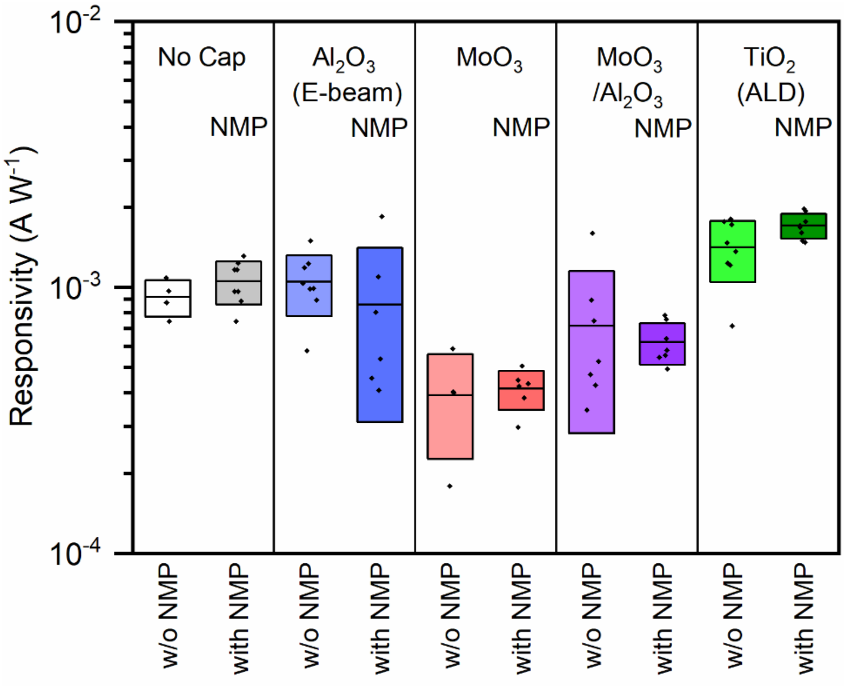

Figure S2. Responsivities of bP photodetectors with different capping layers and NMP/non-NMP bP dispersions under $\lambda = 1.55$ μm illumination at room temperature ($I_{dark} = 50$ μA, $P = 2.8$ mW mm^{-2}, 10 Hz). Each data point is one photodetector and all photodetectors of the same type were fabricated on the same substrate from the same bP thin film. Middle line is average R and box ends are 1 standard deviation.

Supporting Information 3 – MWIR Photoluminescence Measurements

Photoluminescence (PL) of the solution-deposited bP thin films was measured and compared with mechanically exfoliated individual multilayer bP flakes. A λ = 660 nm laser (S1FC660, Thorlab) was focused then incident onto the samples. After the Cassegrain and a longpass filter (cut-on wavelength λ = 2.4 μm, Edmund), the PL signal was focused by a parabolic mirror onto an InSb detector connected to a lock-in amplifier (SR830, Stanford Research Systems). The measurements were performed at a modulated frequency of 5 Hz. At a pumping power of ~3.2 mW, a mechanically exfoliated individual multilayer bP flake was measured to have a PL quantum yield (PLQY) of ~2.4%, which is consistent with thicker bP flakes (>100 nm) reported previously.[6] The PL signals of the fabricated solution-deposited bP thin films were measured at ~1/15 of the mechanically exfoliated bP flakes. For the calibration of PLQY, a λ = 4.5um laser (ITC4002QCL) with known emission power was measured under the same condition to obtain an estimation of the system's collection efficiency.

Supporting Information 4 – Cross-Section of bP Photodetector

Figure S4 is a high-resolution helium ion microscope (HIM) image of the bP photodetector cross-section with ~120 nm mean bP film thickness and ~13 nm Al_2O_3 capping layer. The cross-section was cleaved by scoring the edge of the Si substrate and fracturing the substrate along the horizontal crystal plane. This results in some fraying and bending of the bP film at the cross-section as it was not directly cut/milled. The sample is slightly tilted in the image (not 90° to cross section) to view the top edge of the device.

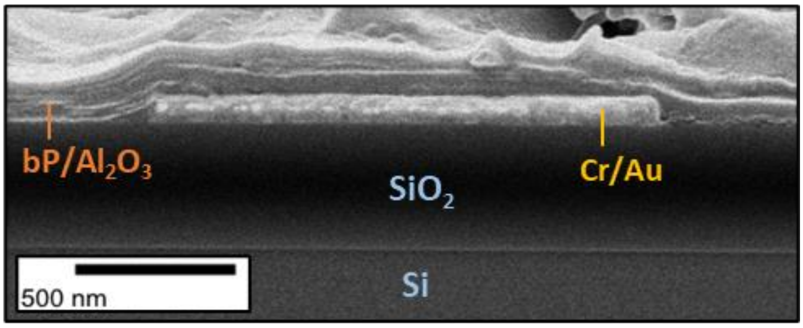

Figure S4. High-resolution HIM image of the bP photodetector cross-section.

Supporting Information 5 – Extended Characterization of bP Photodetectors

The 3-dB roll-off frequency ($f_{\text{-3dB}}$) can be determined from frequency-dependent R or photocurrent measurements at the frequency where $f_{\text{-3dB}} = 0.707 \times f_{\text{0dB}}$. The $f_{\text{-3dB}}$ point for the TiO_2-capped bP photodetector can be obtained from the frequency-dependent R measurements plotted in **Figure 5b** in the main text, and was found to be ~10 Hz at room temperature and ~100 Hz at 78 K. The $f_{\text{-3dB}}$ point for the Al_2O_3-capped bP photodetector could not be obtained from the frequency dependent R measurements plotted in **Figure 5a** in the main text, as it was beyond the modulation frequency limit of the λ = 2.2 μm illumination source. These results were well supported by the separately measured 90-10% rise/fall times displayed in **Figures 5c,d**. Using the relationship $f_{\text{-3dB}} = 0.35/t_R$, the TiO_2-capped bP photodetector response times correspond to an estimated $f_{\text{-3dB}}$ of 14 Hz and 35 Hz at room temperature and 78 K, respectively.

The gain (G) of the Al_2O_3-capped bP photodetector under λ = 2.2 μm at 1 Hz modulation can also be estimated from:

$$G = \frac{I_{ph}\,\mathrm{h}\,f}{P\,Abs\,q},$$

where I_{ph} is photocurrent, f is incident light frequency, P is illumination power, Abs is absorbance of the bP photodetector at the incident light frequency, h is the Planck constant, and q is the elementary charge. For the conditions where D^* is calculated, and using the absorbance of the device at λ = 2.2 μm from the measurement in Figure 3c, G is calculated as,

$$G = \frac{7.96\ \mu\mathrm{A} \times (6.626 \times 10^{-34}\ \mathrm{J\ Hz^{-1}}) \times (1.36 \times 10^{14}\ \mathrm{Hz})}{(0.1\ \mathrm{mW}) \times (0.49) \times (1.602\ \times 10^{-19}\ \mathrm{A\ s})}$$

$$G \ngtr 1$$

A gain value less than unity suggests that G is not a major contributor to responsivity in these devices, which aligns with the relatively fast rise/fall times obtained.

Supporting Information 6 – I_D-V_G and I_D-V_D of bP Photodetectors

We measured the I_D-V_G and I_D-V_D of the fabricated bP photodetectors (120 nm mean bP film thickness) using a substrate back gate. The gate modulation of I_D was small due to the position of the bP film channel being above the IDEs, instead of between the IDEs and back gate (**Figure S6a**). A significant I_D of 200 µA could be achieved with V_D < 1 V (**Figure S6b**).

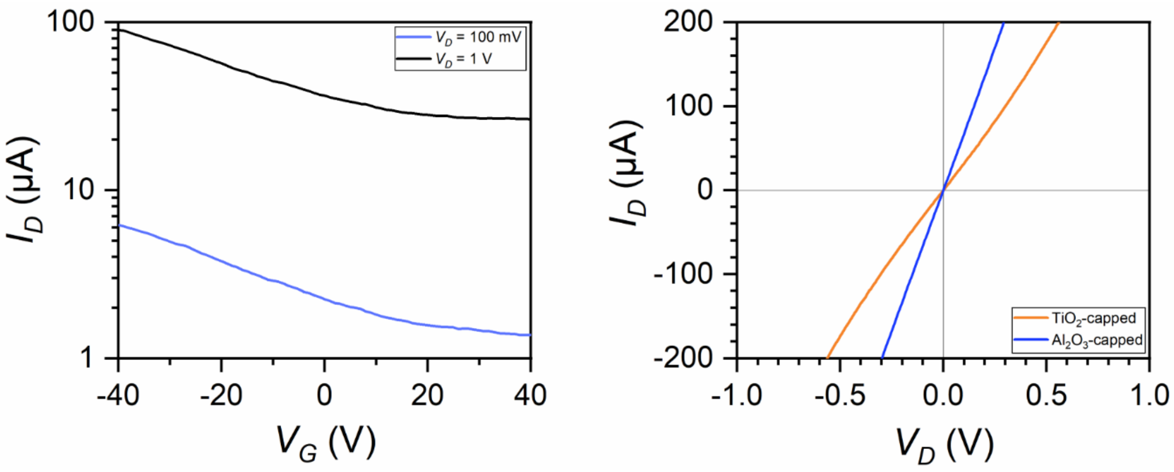

Figure S6. a) I_D-V_G of Al_2O_3-capped bP photodetector at V_D =0.1 and 1 V. **b)** I_D-V_D of bP photodetectors in the dark.

Supporting Information 7 – bP IR Camera Set Up and Aperture/Mask IR Images

Figure S7a presents a schematic of the set up for the bP IR camera to obtain IR shape images. **Figure S7b** middle row shows the photocurrent measured for each pixel to obtain the raw IR images of the aperture (left), mask (middle), and uniform background illumination (right). The average photocurrent of each pixel column (a separate bP photodetector in the linear array) was divided by the uniform illumination power to obtain the relative responsivity of each bP photodetector in the linear array (each pixel column). **Figure S7b** top row then shows the same IR images normalized by the relative responsivity of each bP photodetector in the linear array. **Figure S7b** bottom row also shows the phase difference between the measured and reference modulation frequency, where ~0 phase difference indicates the signal was successfully locked-in by the lock-in amplifier.

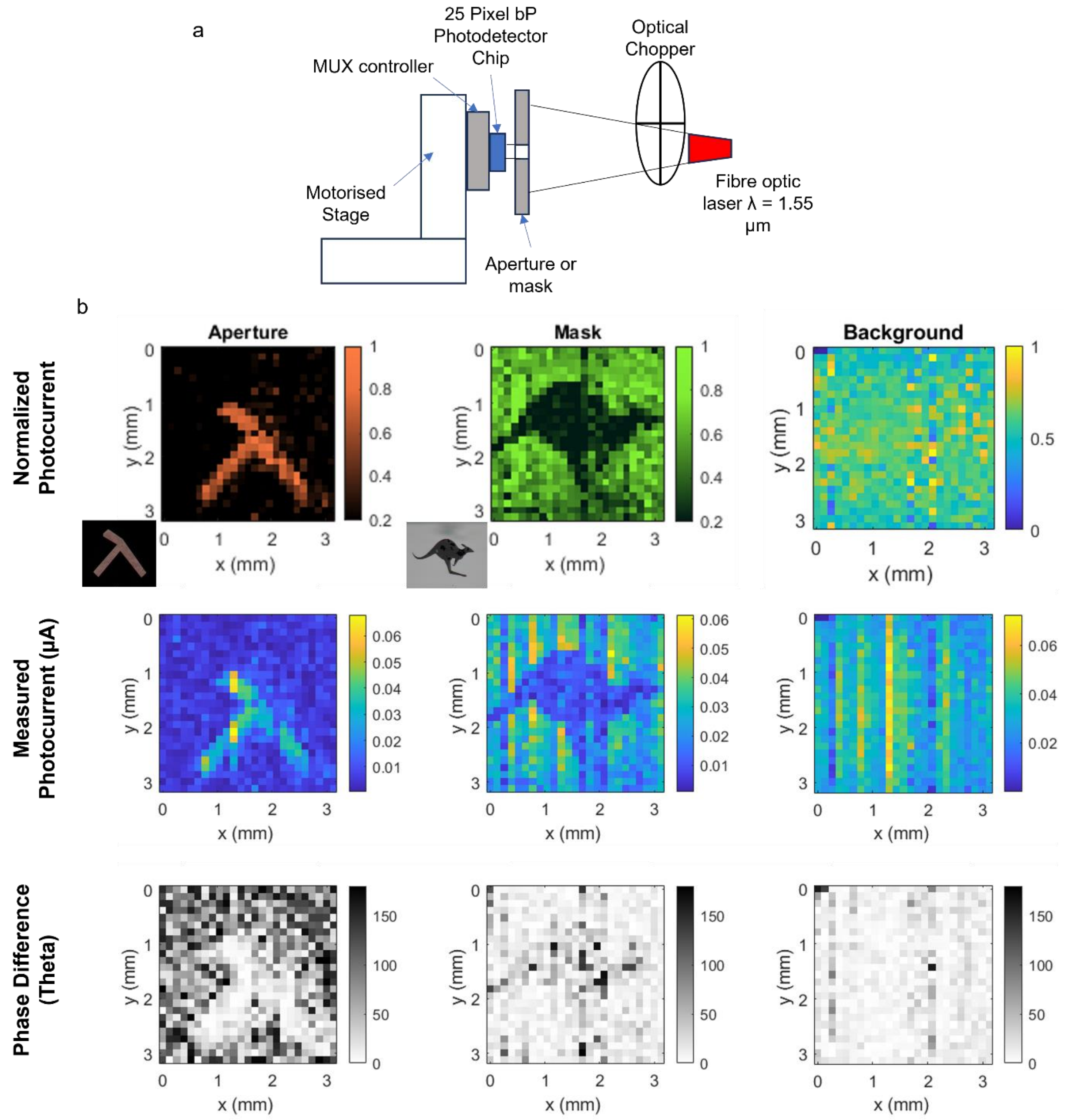

Figure S7. a) Schematic of the bP IR camera set up for obtaining IR images. **b)** IR images with $\lambda = 1.55$ μm illumination obtained from the bP IR camera. Left, middle, and right columns are IR images obtained while using 'λ'-shaped aperture, kangaroo-shaped mask, and no aperture (background), respectively. Top and middle rows are normalized and measured photocurrent IR images obtained, respectively, and bottom row is the lock-in phase difference measured at each pixel.

Supporting Information 8 – bP IR Camera Set Up and Broadband IR Images

Figure S8a presents a schematic of the set up for the bP IR camera to obtain the broadband source IR images. **Figure S8b** bottom row shows the photocurrent measured for each pixel to obtain the raw IR images of the broadband IR light source without filters (left), with the 1.2 µm longpass filter (middle), and the 2 µm bandpass filter (right). **Figure S8b** top row then shows the same IR images normalized by the responsivity of each bP photodetector in the linear array.

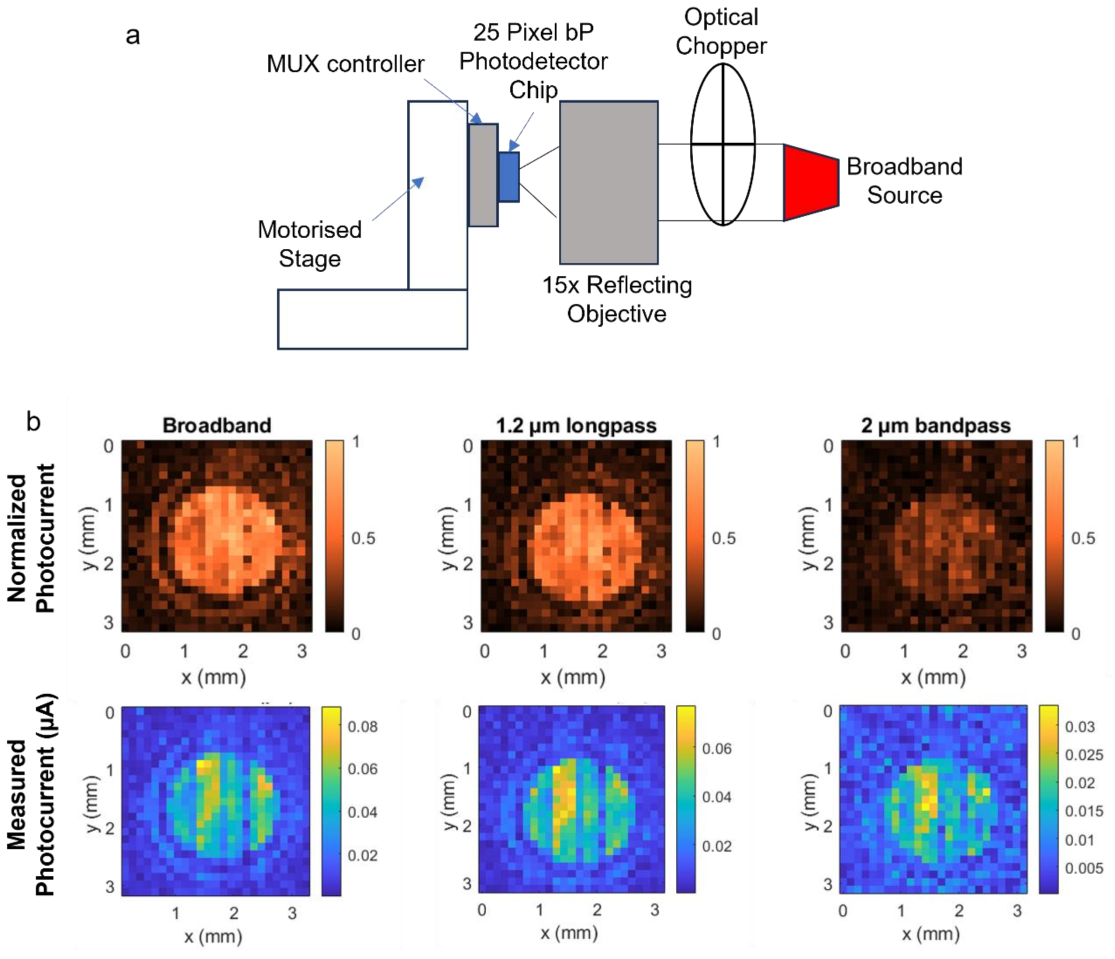

Figure S8. a) Schematic of the bP IR camera set up for obtaining IR images with broadband source. **b)** IR images with 1500 K blackbody broadband IR illumination obtained from the fabricated bP IR linear camera. Left, middle, and right columns are IR images obtained while using no filter, $\lambda = 1.2$ µm longpass filter, and $\lambda = 2$ µm bandpass filter (500 nm FWHM), respectively. Top and bottom rows are normalized and measured photocurrent IR images, respectively.

References

[1] M. Batmunkh, M. Myekhlai, A. S. R. Bati, S. Sahlos, A. D. Slattery, Tania M. Benedetti, V. R. Gonçales, C. T. Gibson, J. J. Gooding, R. D. Tilley, J. G. Shapter, *J. Mater. Chem. A* **2019**, *7*, 12974.

[2] C. Hao, F. Wen, J. Xiang, S. Yuan, B. Yang, L. Li, W. Wang, Z. Zeng, L. Wang, Z. Liu, Y. Tian, *Adv. Funct. Mater.* **2016**, *26*, 2016.

[3] M. Lee, A. K. Roy, S. Jo, Y. Choi, A. Chae, B. Kim, S. Y. Park, I. In, *Nanotechnology* **2017**, *28*, 125603.

[4] D. Xiang, C. Han, J. Wu, S. Zhong, Y. Liu, J. Lin, X.-A. Zhang, W. Ping Hu, B. Özyilmaz, A. H. C. Neto, A. T. S. Wee, W. Chen, *Nat. Commun.* **2015**, *6*, 6485.

[5] M. Bat-Erdene, M. Batmunkh, C. J. Shearer, S. A. Tawfik, M. J. Ford, L. Yu, A. J. Sibley, A. D. Slattery, J. S. Quinton, C. T. Gibson, J. G. Shapter, *Small Methods* **2017**, *1*, 1700260.

[6] N. Higashitarumizu, S. Z. Uddin, D. Weinberg, N. S. Azar, I. K. M. Reaz Rahman, V. Wang, K. B. Crozier, E. Rabani, A. Javey, *Nat. Nanotechnol.* **2023**, *18*, 507.
